# Spatially- and temporally-controlled postnatal p53 knockdown cooperates with embryonic Schwann cell precursor *Nf1* gene loss to promote malignant peripheral nerve sheath tumor formation

**DOI:** 10.18632/oncotarget.7232

**Published:** 2016-02-07

**Authors:** Angela C. Hirbe, Sonika Dahiya, Dinorah Friedmann-Morvinski, Inder M. Verma, D. Wade Clapp, David H. Gutmann

**Affiliations:** ^1^ Division of Medical Oncology, Department of Medicine, Washington University School of Medicine, St. Louis, MO, USA; ^2^ Department of Pathology and Immunology, Washington University School of Medicine, St. Louis, MO, USA; ^3^ The Salk Institute of Biological Studies, Laboratory of Genetics, La Jolla, CA, USA; ^4^ Department of Medical and Molecular Genetics, Indiana University School of Medicine, Indianapolis, IN, USA; ^5^ Department of Neurology, Washington University, St. Louis, MO, USA

**Keywords:** Neurofibromatosis Type 1, MPNST, lentivirus, p53, mouse models

## Abstract

Malignant peripheral nerve sheath tumors (MPNSTs) are highly aggressive sarcomas that arise sporadically or in association with the Neurofibromatosis type 1 (NF1) cancer predisposition syndrome. In individuals with NF1, MPNSTs are hypothesized to arise from *Nf1*-deficient Schwann cell precursor cells following the somatic acquisition of secondary cooperating genetic mutations (*e.g*., p53 loss). To model this sequential genetic cooperativity, we coupled somatic lentivirus-mediated p53 knockdown in the adult right sciatic nerve with embryonic Schwann cell precursor *Nf1* gene inactivation in two different *Nf1* conditional knockout mouse strains. Using this approach, ∼60% of mice with Periostin-Cre-mediated *Nf1* gene inactivation (Periostin-Cre; *Nf1*^flox/flox^ mice) developed tumors classified as low-grade MPNSTs following p53 knockdown (mean, 6 months). Similarly, ∼70% of *Nf1*+/− mice with GFAP-Cre-mediated *Nf1* gene inactivation (GFAP-Cre; *Nf1*^flox/null^ mice) developed low-grade MPNSTs following p53 knockdown (mean, 3 months). In addition, wild-type and *Nf1+/−* mice with GFAP-Cre-mediated *Nf1* loss develop MPNSTs following somatic p53 knockout with different latencies, suggesting potential influences of *Nf1+/−* stromal cells in MPNST pathogenesis. Collectively, this new MPNST model system permits the analysis of somatically-acquired events as well as tumor microenvironment signals that potentially cooperate with *Nf1* loss in the development and progression of this deadly malignancy.

## INTRODUCTION

MPNSTs are an aggressive subtype of soft-tissue sarcoma that develops in association with peripheral nerves or nerve roots. Composed of neoplastic Schwann cells, these malignancies are thought to arise from benign nerve sheath tumors, termed plexiform neurofibromas. Plexiform neurofibromas are frequently detected during early childhood, raising the possibility that they are congenital tumors that initiate during fetal or early postnatal life. While transformation of these plexiform neurofibromas in childhood is uncommon, the development of pain or motor weakness associated with a pre-existing plexiform neurofibroma in a teenager or young adult should prompt evaluation for a MPNST. In patients with these malignant tumors, overall survival is poor, and successful treatment options are limited. Even when surgery is employed in combination with radiation or chemotherapy, ∼50% of individuals will experience local recurrence as well as distant metastases, and the majority will succumb to their cancer within 5 years [1-3].

MPNSTs occur most frequently in the setting of the Neurofibromatosis 1 (NF1) tumor predisposition syndrome, affecting approximately 8-13% of people with NF1 [4]. Individuals with NF1 are born with a germline mutation in one copy of the *NF1* gene, such that all cells in their bodies have one dysfunctional *NF1* allele. However, tumorigenesis requires somatic loss of the other *NF1* allele in the appropriate cell of origin. Consistent with a key role for the *NF1* gene in both NF1-associated and sporadic MNPST pathogenesis, bi-allelic *NF1* gene inactivation has been reported in approximately 60-90% of NF1-associated MPNSTs and 40-60% of sporadic cases [5, 6]. While loss of *NF1* gene expression is required for MPNST development, it is not sufficient. As such, MPNST formation requires additional cooperating genetic events, the most frequent of which is mutational inactivation of the *TP53* tumor suppressor gene, occurring in approximately 75% of cases [7-9].

In this regard, genetically-engineered mouse (GEM) lines with conditional *Nf1* gene inactivation in Schwann cell precursors do not develop MPNSTs [10-14] unless coupled with concomitant *Trp53* loss [15, 16], epidermal growth factor receptor (*Egfr*) amplification [7, 17], *Pten* loss [18], or *Ink4a* deletion [19, 20]. In each of these GEM strains, the cooperating genetic mutation was introduced simultaneously with Schwann cell precursor *Nf1* gene inactivation throughout the body. While each of these models has been informative for understanding MPNST pathogenesis, they do not permit temporal (timing of the cooperating somatic genetic change) or spatial (specific nerve location) control over MPNST development. To generate a model in which temporal control of the transforming genetic alteration can be achieved in a single nerve location, we employed two GEM strains in which Cre-mediated *Nf1* inactivation occurs in Schwann cell precursor cells during embryogenesis and p53 knockdown is somatically acquired at 6-8 weeks of age in cells within the right sciatic nerve. Using this approach, 60-70% of mice develop sciatic nerve MPNSTs associated with focal neurological dysfunction. The coupling of somatic retroviral knockdown and embryonic *Nf1* gene inactivation establishes an experimentally-manipulable platform to evaluate other cooperating genetic changes in MPNST pathogenesis as well as preclinical mouse strains in which clinical symptomatology can be used to monitor tumor progression.

## RESULTS AND DISCUSSION

Leveraging whole exome sequencing methodologies, a recent study from our laboratory revealed a temporal sequence of genetic changes in a single patient with progression of an NF1-associated plexiform neurofibroma to MPNST over a 14-year period. Analysis of these pathological specimens demonstrated an increasing proportion of cells with a somatic *NF1* gene mutation (second hit) as the tumor progressed from a benign plexiform neurofibroma to a MPNST. In addition, loss of one copy of the *TP53* gene was only detected at the MPNST stage [21]. These observations in a single patient support a model in which *NF1* gene inactivation precedes *TP53* mutation in the pathogenesis of NF1-associated MPNST.

To model this temporal sequence in mice, we leveraged two different GEM strains in which bi-allelic *Nf1* gene inactivation occurred in Schwann cell precursors during embryonic development. For these experiments, *Nf1*^flox/flox^ mice were intercrossed with either Periostin-Cre [22, 23] or GFAP-Cre [24] mice to eliminate *Nf1* protein (neurofibromin) expression in Schwann cell precursors. We first sought to generate mice with a germline null *Nf1* gene mutation and a conditional *Nf1* allele; however, the majority of Periostin-Cre; *Nf1*^flox/null^ mice did not survive to weaning age (∼3-4 weeks), as previously reported by others [23]. As such, Periostin-Cre; *Nf1*^flox/flox^ mice and GFAP-Cre; *Nf1*^flox/null^ mice, were used for these experiments. Importantly, neither strain develops MPNSTs without the introduction of additional genetic alterations (*e.g*., somatic *p53* knockdown).

**Figure 1:**
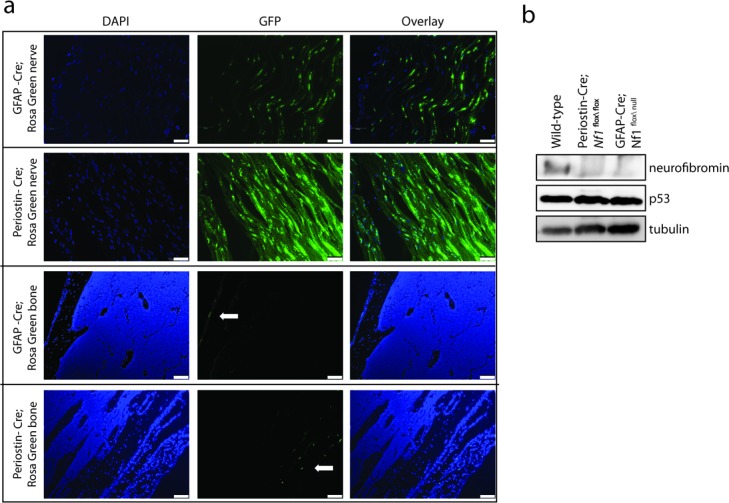
Murine MPNST model system employed **a.** Representative images of sciatic nerves and femurs (bone) from GFAP-Cre (FVB-Tg(GFAP-cre)25Mes/J) and Periostin-Cre mice crossed to Rosa-Green reporter mice. Green fluorescent protein (GFP)-positive cells were observed in the sciatic nerve and bone periosteum (white arrows), but not in the hematopoietic cells within the bone parenchyma. Scale bar, 50μm. **b.** Western blotting reveals complete loss of neurofibromin expression and intact p53 expression in Periostin-Cre; *Nf1*^flox/flox^ and GFAP-Cre; *Nf1*^flox/null^ sciatic nerves compared to intact neurofibromin and p53 expression in the wild-type sciatic nerve.

In the Periostin-Cre model, promoter activity is detected as early as embryonic day 10 (E10) within post-migratory Schwann cell progenitor cells. Based on published reporter activity studies, recombination occurs in the enteric nervous system, peripheral nervous system (including Schwann cells), and within a subpopulation of cardiac outflow tract mesenchymal cells [22]. As expected, intercrossing Periostin-Cre mice with a Rosa-GREEN reporter strain [25] revealed green fluorescent protein (GFP) expression in sciatic nerves (Figure 1a). Similarly, in the GFAP-Cre model, where promoter activity has been reported as early as E13.5 in the brain [24], we observed robust GFP expression in the sciatic nerves of adult GFAP-Cre mice following intercrossing with Rosa-GREEN reporter mice (Figure 1a).

To determine whether Cre-mediated excision might also occur in the hematopoietic cells that populate the tumor microenvironment [12, 26-28] (*e.g*., mast cells and macrophages), both Cre driver lines were intercrossed with Rosa-GREEN reporter mice [25]. Whereas GFP expression was observed within the adult sciatic nerve, there was no GFP labeling within the bone marrow (Figure 1a). The lack of GFP expression in the bone parenchyma demonstrates that Cre-mediated excision is largely limited to the Schwann cell precursors within the sciatic nerve, rather than infiltrating bone marrow-derived stromal cells in the tumor microenvironment.

Western blot analysis of the sciatic nerves of Periostin-Cre; *Nf1*^flox/flox^ mice revealed loss of neurofibromin expression, but intact p53 expression (Figure 1b). The absence of neurofibromin expression demonstrates that early *Nf1* gene inactivation in Schwann cell precursors occurs prior to the induction of shRNA retrovirus-mediated *p53* knockdown. Similar to Periostin-Cre; *Nf1*^flox/flox^ mice, no neurofibromin expression was detected in the sciatic nerves of GFAP-Cre; *Nf1*^flox/null^ mice by western blot (Figure 1b).

**Figure 2:**
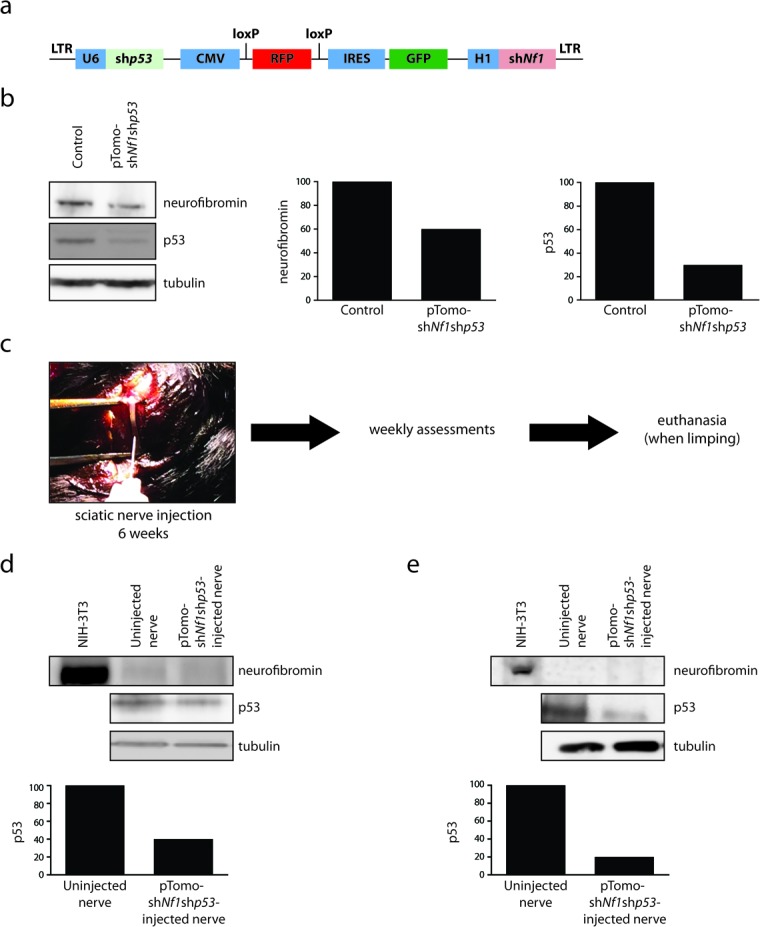
Lentiviral introduction of pTomo-sh*Nf1*;sh*p53* into the sciatic nerve **a.** Schematic of the pTomo-sh*Nf1*;sh*p53* lentivirus construct used in these studies [29]. **b.** Western blotting demonstrates a 40% reduction in neurofibromin expression and a 70% reduction in p53 expression 96 hours after pTomo-sh*Nf1*;sh*p53* lentiviral infection of NIH-3T3 cells. **c.** Schematic of study design. **d.** Western blotting reveals complete loss of neurofibromin expression coupled with a 60% reduction in p53 expression within the sciatic nerves of Periostin-Cre; *Nf1*^flox/flox^ mice injected with pTomo-sh*Nf1*;sh*p53* virus relative to uninjected control nerves. **e.** Western blotting reveals complete loss of neurofibromin expression coupled with an 80% reduction in p53 expression within the sciatic nerves of GFAP-Cre; *Nf1*^flox/flox^ mice injected with pTomo-sh*Nf1*;sh*p53* virus relative to uninjected control nerves.

Next, to introduce p53 knockdown specifically in cells within the sciatic nerve, we leveraged a lentiviral approach in which *Nf1* and *Trp53* knockdown occurs following *p53* shRNA virus injection (Figure 2a). This construct has previously been employed to reduce *Nf1* and *Trp53* gene expression in the brain *in vitro* and *in vivo*, resulting in high-grade gliomas [29]. In this regard, we observed 40% and 70% reductions in neurofibromin and p53 expression, respectively, in NIH-3T3 cells 96 hours post-lentiviral infection *in vitro* (Figure 2b). The choice to employ a lentivirus containing shRNAs targeting both the *Nf1* and *Trp53* genes reflected a lack of reproducible knockdown (KD) when the identical *Trp53* shRNA was employed alone (data not shown). Importantly, since *Nf1* expression has already been silenced in GFAP-Cre; *Nf1*^flox/null^, GFAP-Cre; *Nf1*^flox/flox^, and Periostin-Cre; *Nf1*^flox/flox^ mice, the effects of shRNA *Nf1* KD on the preneoplastic Schwann cell component should be negligible.

For all MPNST induction experiments, adult sciatic nerves were surgically isolated and injected with *p53* shRNA lentivirus when the mice reached 6-8 weeks of age. Mice were then monitored by serial examination until they demonstrated neurological abnormalities (*e.g*., right leg weakness), at which time they were euthanized for gross pathological and histological analyses (Figure 2c).

**Figure 3:**
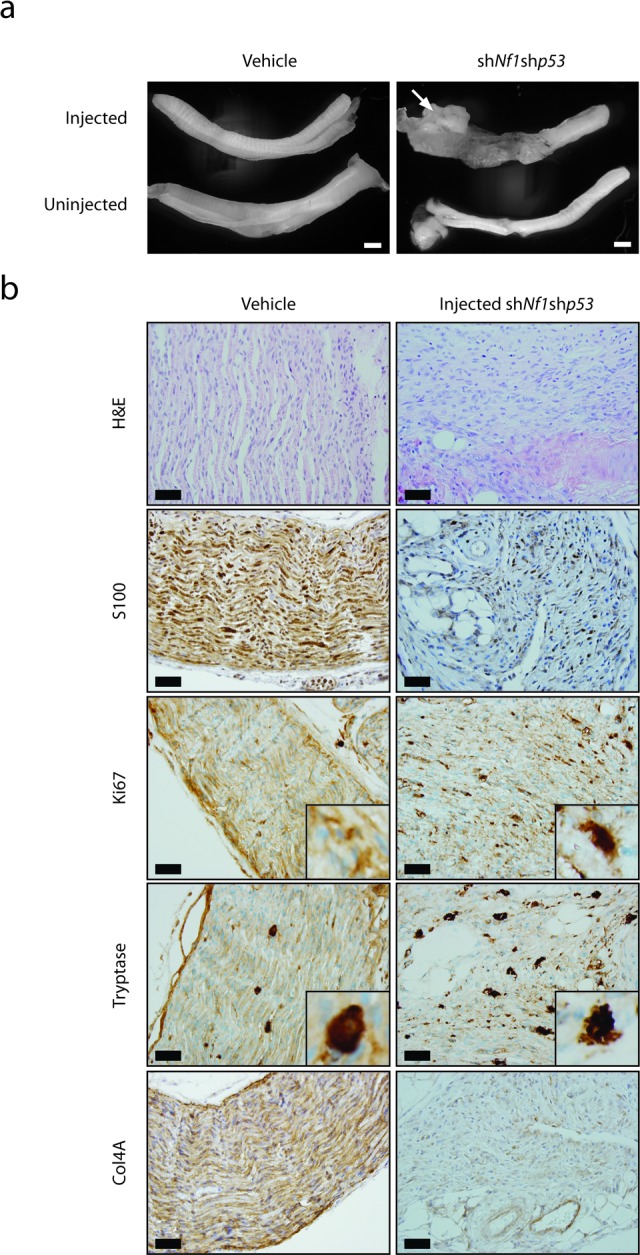
MPNST development in mice with embryonic Schwann cell precursor *Nf1* loss and postnatal somatic *Trp53* reduction **a.** Gross images of the sciatic nerves from Periostin-Cre; *Nf1*^flox/flox^ mice injected with vehicle or pTomo-sh*Nf1*;sh*p53* virus. Low-grade MPNSTs were only observed in the sciatic nerves of Periostin-Cre; *Nf1*^flox/flox^ mice injected with pTomo-sh*Nf1*;sh*p53* virus. Scale bar, 1000μm. **b.** Sciatic nerve sections from Periostin-Cre; *Nf1*^flox/flox^ mice injected with pTomo-sh*Nf1*;sh*p53* virus demonstrate increased cellularity, nuclear pleiomorphism, and mitotic figures, consistent with low-grade MPNST (H&E). Induced MPNSTs exhibit reduced S100 β-staining, increased Ki67 labeling, increased mast cell infiltration (tryptase staining), and collagen-4A (Col4A) basement membrane immunoreactivity. Scale bar, 40μm.

Initial experiments used mice in which neurofibromin expression was ablated in periostin-expressing cells, including Schwann cell precursors within the peripheral nerves (Periostin-Cre; *Nf1*^flox/flox^ mice), consistent with previous lineage tracing studies [30]. While the precise cell of origin is not known in this model, Cre-driven reporter expression has been observed in brain lipid binding protein (BLBP)-immunoreactive cells, which represent Schwann cell precursors or immature Schwann cells [30]. To induce MPNSTs, pTomo-sh*Nf1*;sh*p53* lentivirus or vehicle was injected into the right sciatic nerves of 6-8 week old Periostin-Cre; *Nf1*^flox/flox^ mice, resulting in GFP expression following Cre-mediated recombination (Supplemental Figure 1a). Following the injection of this lentivirus into the right sciatic nerves of 6-8 week old mice, the pre-existing complete loss of neurofibromin expression in these nerves was now accompanied by a 60% reduction in p53 protein expression within the tumors (Figure 2d). With an average latency of 183 days post-injection (Table 1), ∼60% of the pTomo-sh*Nf1*;sh*p53* lentivirus-injected Periostin-Cre; *Nf1*^flox/flox^ mice exhibited paresis of the affected leg, as evidenced by pronounced right-sided lower extremity limping. At this time, these mice were euthanized and their sciatic nerves evaluated for the presence of a malignant tumor. In 10/18 of these mice, grossly-appearing mass lesions were appreciated in the nerves of lentiviral-injected animals, but not in the vehicle-injected controls (Figure 3a).

**Table 1:** MPNST development in Nf1 mutant mice following p53 knockdown

Mouse genotype (injection)	Hyperplasia	MPNST	MPNST latency, mean days (range)
Periostin-Cre; *Nf1*^flox/flox^ (vehicle)	2/11	0/11	No tumors: 83-304d*
Periostin-Cre; *Nf1*^flox/flox^ (pTomo-sh*Nf1*;sh*p53*)	8/18	10/18	183d (144-278d)
GFAP-Cre; *Nf1*^flox/null^ (vehicle)	2/6	0/6	No tumors: 49-217d*
GFAP-Cre; *Nf1*^flox/null^ (pTomo-sh*Nf1*;sh*p53*)	3/11	8/11	91d (25-211d)
GFAP-Cre; *Nf1*^flox/flox^ (vehicle)	0/4	0/4	No tumors:129-153d*
GFAP-Cre; *Nf1*^flox/flox^ (pTomo-sh*Nf1*;sh*p53*)	2/5	3/5	176d (117-298d)
Wild-type (pTomo-sh*Nf1*;sh*p53*)	0/4	0/4	No tumors: 182-365d^#^
*Nf1*+/− (pTomo-sh*Nf1*;sh*p53*)	0/4	0/4	No tumors: 182-365d^#^

* Vehicle mice were euthanized and analyzed at time points equivalent to those when *p53* shRNA-injected mice were euthanized or at a time when they became ill due to non-tumor-related issues (rectal prolapse or hydrocephalus). The details are provided below:
Vehicle-injected GFAP-Cre; *Nf1*^flox/null^ mice: 2 mice at 49 days, 2 mice at 154 days and 2 mice at 217 days. Vehicle-injected Periostin-Cre; *Nf1*^flox/flox^ mice: 1 mouse at 83 days, 3 mice at 144 days, 2 mice at 154 days, 2 mice at 230 days, 2 mice at 259 days, and 1 mouse at 304 days. Vehicle-injected GFAP-Cre; *Nf1*^flox/flox^ mice: 2 mice at 129 days, 1 mouse at 138 days, and 1 mouse at 158 days. Wild-type and *Nf1*+/− mice: 2 mice at 183 days and 2 mice at 365 days for each group.

# Wild-type and *Nf1*+/− mice were euthanized and analyzed at 6 month or 12 month time points, as no mice developed symptoms or non-tumor-related issues.

Neuropathological analyses of the resulting tumors revealed striking hypercellularity, mild to moderate nuclear pleiomorphism, reduced S100β immunopositivity, increased Ki67 labeling (Figure 3b), infiltration of adjacent fibroadipose tissue, and mitotic figures (Supplemental Figure 1b), as seen in their human low-grade MPNST counterparts. Additionally, these murine MPNSTs retained basement membrane staining (Collagen 4A; Col4a immunoreactivity) and demonstrated mast cell infiltration visualized by tryptase staining, as reported in other murine MPNST-like tumors [31] (Figure 3b). Based on the observed increased cellularity, nuclear atypia, and mitotic activity, these lesions are most accurately classified as low-grade MPNST, as suggested by the Comparative Pathology of Nerve Sheath Tumors in Mouse Models and Humans Committee Consensus Report [32]. The remaining 8 mice exhibited hyperplasia only, similar to some of the vehicle-injected mice.

The development of MPNSTs in mice without complete loss of p53 expression (Figure 2d) suggests that bi-allelic (complete) inactivation of the *Trp53* gene might not be necessary for malignant transformation. While we cannot exclude the acquisition of additional genetic changes that effectively abrogate p53 signaling, previous reports in both mouse models and human pathological MPNST specimens have likewise support the concept that *TP53* haploinsufficiency may be sufficient for MPNST formation [7, 9]. Similarly, our recent whole exome sequencing analysis of a single patient with NF1-related plexiform neurofibroma malignant transformation revealed only heterozygous *TP53* loss [21].

The development of MPNSTs in Periostin-Cre; *Nf1*^flox/flox^ mice raises the intriguing possibility that malignant transformation does not require heterozygous *Nf1* loss in cells within the tumor microenvironment. While previous studies from one of our laboratories (D.W.C.) have clearly demonstrated a critical role for infiltrating *Nf1*+/− stromal cells in benign murine plexiform neurofibroma formation and growth [12, 27, 28], the apparent stromal independence in MPNST pathogenesis could reflect differences between low-grade tumor growth requirements and those operative in malignant cancers. In this respect, similar differences have also been reported for murine *Nf1* brain tumors: *Nf1*+/− stromal cells are required for low-grade murine optic glioma formation and continued growth *in vivo* [33-35], but high-grade glioblastoma development can occur following *Nf1* and *p53* inactivation in nestin^+^ neural stem cells alone *in vivo* [36].

**Figure 4:**
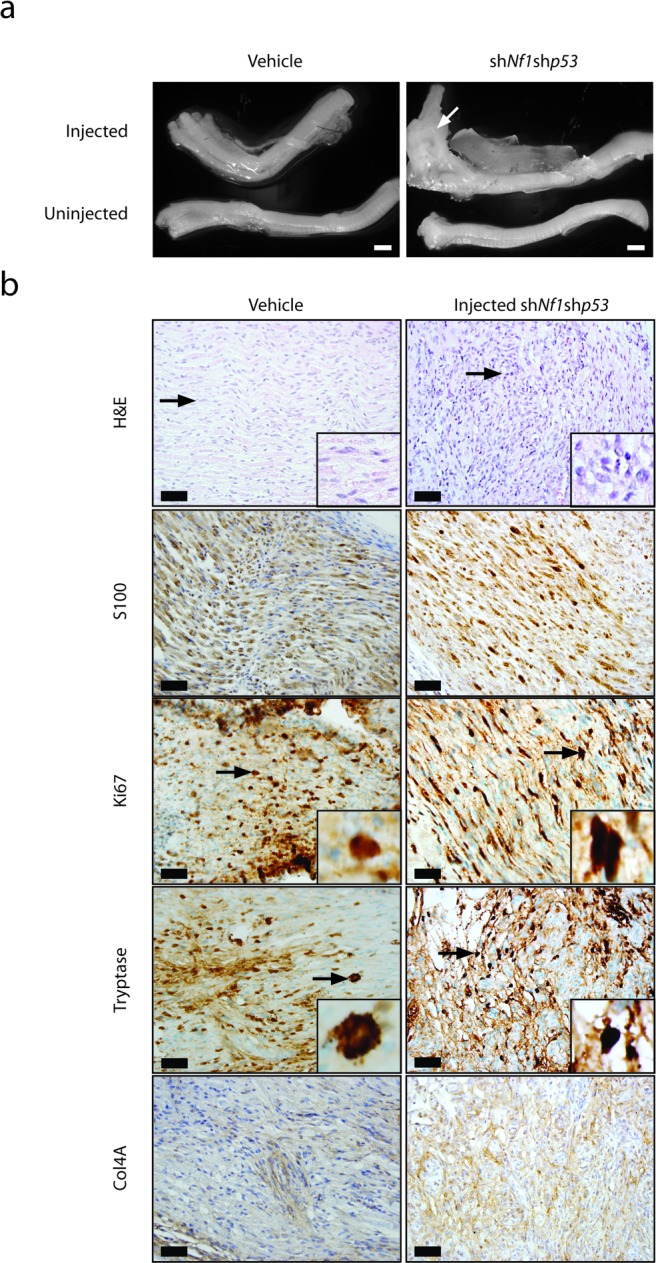
MPNST formation is accelerated in *Nf1+/−* mice harboring embryonic *Nf1* loss and somatic *Trp53* reduction **a.** Gross images of the sciatic nerves from GFAP-Cre; *Nf1*^flox/null^ mice injected with vehicle or pTomo-sh*Nf1*;sh*p53* virus. Low-grade MPNSTs were only observed in the sciatic nerves of GFAP-Cre; *Nf1*^flox/null^ mice injected with pTomo-sh*Nf1*;sh*p53* virus. Scale bar, 1000μm. **b.** Sciatic nerve sections from GFAP-Cre; *Nf1*^flox/null^ mice injected with pTomo-sh*Nf1*;sh*p53* virus demonstrate increased cellularity and nuclear pleiomorphism, consistent with low-grade MPNST (H&E). Induced MPNSTs exhibit reduced S100 β-staining, increased Ki67 labeling, increased mast cell infiltration (tryptase staining), and collagen-4A (Col4A) basement membrane immunoreactivity. Scale bar, 40μm.

Since the early lethality (pre-weaning) observed in *Nf1*+/− mice with periostin-Cre-mediated *Nf1* gene inactivation precluded an examination of the contribution of the *Nf1*+/− tumor microenvironment to MPNST biology, we sought to reduce the latency of MPNST development by coupling somatic retrovirus-mediated p53 knockdown and embryonic Schwann cell precursor *Nf1* gene inactivation in the setting of an *Nf1*+/− microenvironment. For these experiments, we employed GFAP-Cre mice [24], in which Cre-mediated recombination occurs in the sciatic nerve (Figure 1a). In contrast to the Periostin-Cre; *Nf1*^flox/null^ mice, the majority of which die by 4 weeks of life, *Nf1*^flox/null^; GFAP-Cre mice are viable into adulthood. Similar to the experiments performed with the Periostin-Cre; *Nf1*^flox/flox^ mice, pTomo-sh*Nf1*;sh*p53* lentivirus or vehicle was injected into the sciatic nerves of 6-8 week old GFAP-Cre; *Nf1*^flox/null^ mice. Following the injection of this lentivirus into the right sciatic nerves of 6-8 week old mice, the pre-existing complete loss of neurofibromin expression in these nerves was now accompanied by an 80% reduction in p53 protein expression within the tumors (Figure 2e). While none of the vehicle-injected mice exhibited any neurological deficits, eight of eleven GFAP-Cre; *Nf1*^flox/null^ mice injected with pTomo-sh*Nf1*;sh*p53* lentivirus exhibited right leg weakness. Following euthanasia, gross morphological features of MPNST were observed (Figure 4a). As observed with the pTomo-sh*Nf1*;sh*p53* lentivirus-injected Periostin-Cre; *Nf1*^flox/flox^ mice, MPNSTs in the GFAP-Cre; *Nf1*^flox/null^ mice with somatic p53 knockdown exhibited striking hypercellularity, mild to moderate nuclear pleiomorphism, reduced S100β immunoreactivity, increased Ki67 labeling, mast cell infiltrates (Tryptase staining), infiltration of adjacent fibroadipose tissue, and Col4A immunoreactivity (Figure 3b). However, in contrast to pTomo-sh*Nf1*;sh*p53* lentivirus-injected Periostin-Cre; *Nf1*^flox/flox^ mice, the average time to MPNST formation was only ∼90 days as opposed to ∼183 days.

It is possible that the shortened MPNST latency observed in injected GFAP-Cre; *Nf1*^flox/null^ mice reflected growth-promoting contributions from the heterozygous tumor microenvironment. Support for this idea derives from early findings in GFAP-Cre; *Nf1*^flox/flox^ mice (harboring wild-type stromal cells) injected with pTomo-sh*Nf1*;sh*p53* lentivirus. In this respect, the 3 mice that developed MPNSTs exhibited longer latencies than observed in GFAP-Cre; *Nf1*^flox/null^ mice (average of 176 days, Table 1). However, in striking contrast, 50% of the GFAP-Cre; *Nf1*^flox/null^ mice injected with pTomo-sh*Nf1*;sh*p53* lentivirus had developed a limp and exhibited low-grade MPNST on pathological examination by 50 days post-injection. Coupled with the results obtained using Periostin-Cre; *Nf1*^flox/flox^ mice, it is most likely that cells in the *Nf1*+/− tumor microenvironment provide additional growth factors or chemokines (e.g., CXCL12 [37]) that accelerate the growth of the newly-formed MPNST, leading to a reduced time to clinical symptomatology.

Since the lentivirus employed contained shRNAs that targeted both the *Trp53* and *Nf1* genes, we next sought to determine whether *Nf1* shRNA-mediated KD was sufficient to generate MPNSTs in wild-type or *Nf1+/−* mice. Following injection of pTomo-sh*Nf1*;sh*p53* lentivirus at 6 weeks of age, mice were examined 6 months or 12 months later. No mice developed a limp and no tumors were appreciated on gross or histologic examination (Table 1). These findings lend further support to the hypothesis that loss of *Nf1* gene expression in embryonic Schwann cell precursors is required for plexiform neurofibroma development and subsequent MPNST formation, as previously demonstrated by others [38-40]

The high frequency of MPNSTs in individuals with NF1 coupled with genetic analyses of human MPNSTs argues that *NF1* loss is an obligate genetic event in MPNST development. Moreover, the fact that bi-allelic inactivation of the *NF1* gene is observed in most NF1-associated [5, 6, 41] and sporadic [42] MNPSTs supports a critical role for this tumor suppressor gene in MPNST pathogenesis. While neurofibromin loss in cells of the Schwann cell lineage is required for MPNST formation, it is clearly not sufficient. This lack of sufficiency is nicely illustrated in *Nf1* GEM strains, where mice with conditional *Nf1* gene inactivation in Schwann cell precursors alone also do not develop MPNSTs [10-14], including the GFAP-Cre; *Nf1^flox/null^* and Periostin-Cre; *Nf1*^flox/flox^ mouse strains used in the current study. However, the co-existence of other cooperating genetic events in combination with *Nf1* loss does result in MPNST formation (summarized in Supplemental Table 1). In most of these mouse models, complex intercrosses of transgenic and conditional knockout strains are required, and there is no control over the timing of the acquired somatic genetic event or the location in which the malignancy will arise [7, 15-20, 43-45]. Using a combination of conditional knockout mice and lentivirus-mediated somatic p53 inactivation, we describe an efficient platform for the generation of MPNSTs. As such, this model is ideally suited to define the biological importance of somatically-acquired cooperating genetic events to MPNST pathogenesis, thus allowing investigators to compare the effects of known somatic genetic alterations (e.g., *p53* versus *p16* inactivation) or those identified in large-scale genomic discovery efforts [46]. In addition, this experimental system provides both spatial and temporal control. Future uses of this platform provide opportunities to determine the impact of introducing cooperating genetic mutations as a function of mouse age or in different peripheral nerves. Moreover, since somatic p53 knockdown is directed to the sciatic nerve, these mice develop a neurologic deficit (limp), similar to some patients with MPNSTs. The ability to monitor clinical signs will be important if these models are employed for preclinical studies.

In summary, the implementation of this experimental MPNST model system has allowed us to make two important observations. First, we demonstrate that somatic reduction, but not absence, of *Trp53* expression following *Nf1* gene inactivation is sufficient for MPNST formation. Second, we demonstrate that MPNST formation can occur in the absence of a microenvironment composed of cells heterozygous for a germline *Nf1* gene mutation. Future studies employing this platform may facilitate more rapid analyses of the contributions of other cooperating events to MPNST pathogenesis, the role of the tumor microenvironment in MPNST growth, and the development of radiologic and molecular biomarkers of malignant transformation.

## MATERIALS AND METHODS

### Mice

GFAP-Cre (FVB-Tg(GFAP-cre)25Mes/J) and Rosa-Green reporter mice were purchased from the Jackson Laboratories [24, 25]. Periostin-Cre [22] and *Nf1*^flox/flox^ [47] mice have previously been described. All mice were maintained on a C57Bl/6 background and used in accordance with approved animal studies protocols at the Washington University School of Medicine.

### Lentivirus generation

The pTomo-sh*Nf1*;sh*p53* lentivirus construct used has been previously described [29]. Viral particles were produced by the Viral Vectors Core Facility of the Hope Center for Neurological Diseases at the Washington University School of Medicine.

### Sciatic nerve injections

The right flanks of 6-8 week old mice were shaved and prepped with betadine. The right sciatic nerves were surgically isolated while mice were under anesthesia. 10μl of pTomo-sh*Nf1*;sh*p53* lentivirus (5×10^6^ IU) or 10μl vehicle was injected into the right sciatic nerves of mice using a10μl Hamilton syringe equipped with a 33-gauge needle. The surgical incision was closed with Vetbond and sutured. Mice were followed and examined three times per week until right-sided limping was observed. At this time, mice were euthanized with carbon dioxide and the sciatic nerves isolated for gross and histologic analysis.

### Tissue preparation

For eight-week-old GFAP-Cre; Rosa Green and Periostin-Cre; Rosa Green mouse nerves and bones were Formalin-fixed and paraffin-embedded. 5 micron-thick sections were generated for analysis. Tissues were subsequently dehydrated and coverslipped with Vectashield mounting media containing DAPI (Vector Laboratories).

### Immunohistochemistry

Nerves were processed as described above using primary (Supplemental Table 2) and horseradish peroxidase-conjugated secondary antibodies (Vector Laboratories, Burlingame, CA, USA) in combination with Vectastain Elite ABC development.

### Microscopy

For eight-week-old GFAP-Cre; Rosa Green and Periostin-Cre; Rosa Green mouse nerves and bones, images were acquired at 100x magnification on a Nikon Eclipse TE300 fluorescence inverted microscope equipped with an optical camera (Leica DFC 3000G) and analyzed using Leica Application Suite Advanced Fluorescence 3.20.9652. Seven day post-injection images were acquired at 200x magnification on a Nikon Eclipse TE300 fluorescence inverted microscope equipped with an optical camera (Leica DFC 3000G). Images from tumor bearing mice were acquired at 400x magnification using an Olympus BX51 camera.

### Western blotting

Cell pellets, sciatic nerves, or tumors dissected from sciatic nerves were lysed in buffer containing 1% NP-40 (nonylphenoxypolyethoxylethanol) supplemented with protease inhibitors. Western blotting was performed as previously described [48]. Antibodies used included neurofibromin (Santa Cruz Biotechnology; dilution 1:100), p53 (Cell Signaling; dilution 1:1000), and α-tubulin (Sigma; dilution 1:10000). Densitometry was measured using Life Science Software from UVP VisionWorks LS Version 8.1.1 Image Acquisition and Analysis Software system. α-tubulin serves as an internal protein loading control.

## SUPPLEMENTARY MATERIAL FIGURE AND TABLES


