# Supplementary material for: Spatially- and temporally-controlled postnatal p53 knockdown cooperates with embryonic Schwann cell precursor Nf1 gene loss to promote malignant peripheral nerve sheath tumor formation
Source: Oncotarget. 2016 Feb 7;7(7):7403–14. doi: 10.18632/oncotarget.7232 (PMC4884927; doi:10.18632/oncotarget.7232)
Supplement: Supplementary file 1 [file oncotarget-07-7403-s001.pdf]

Spatially- and temporally-controlled postnatal p53 knockdown cooperates with embryonic Schwann cell precursor *Nf1* gene loss to promote malignant peripheral nerve sheath tumor formation

Supplementary Material

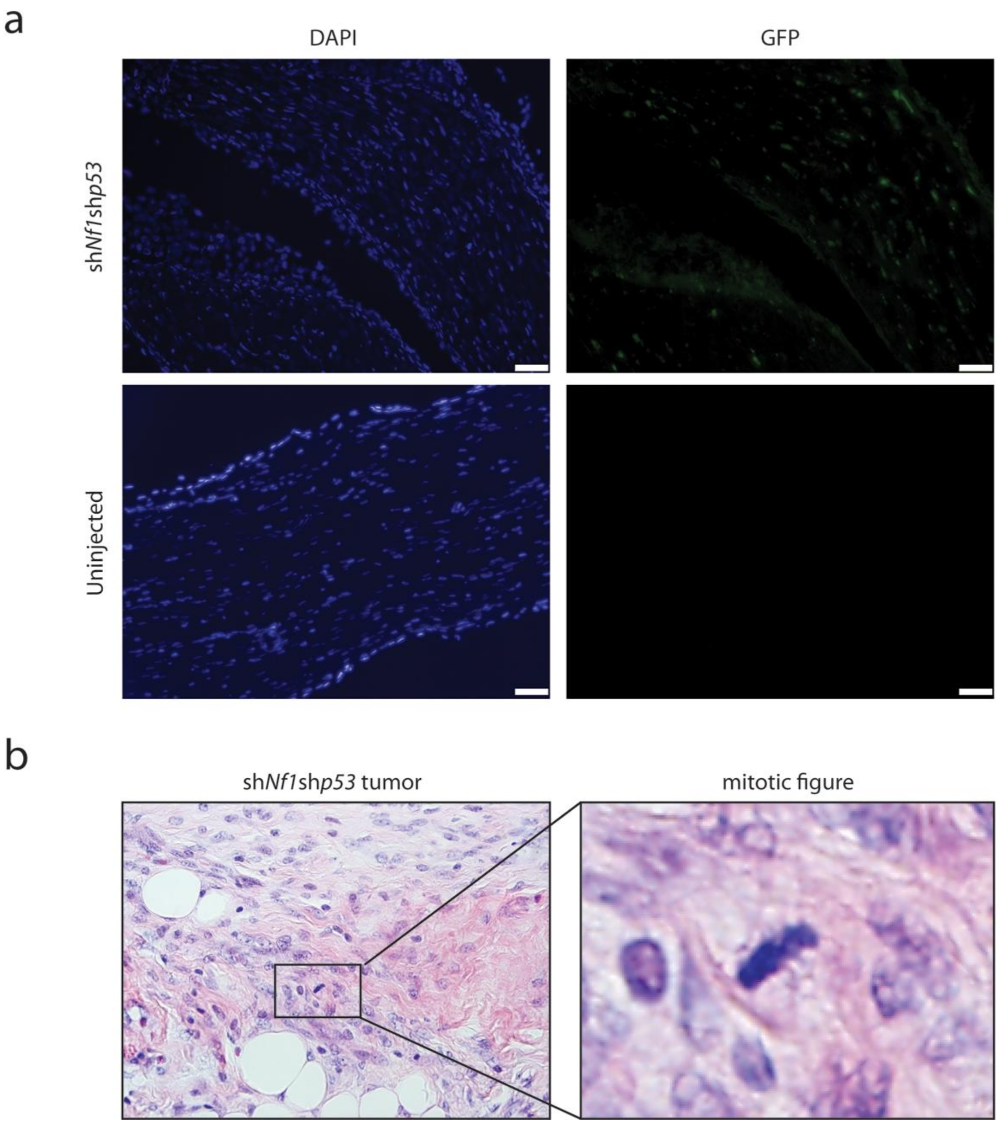

Supplemental Figure 1. (**a**) GFP-positive cells are apparent seven days after the injection of pTomo-sh*Nf1*;sh*p53* virus into the right sciatic nerve. 50μm scale bar. (**b**) Representative image of mitotic figures in a MPNST arising in a Periostin-Cre; *Nf1*$^{flox/flox}$ mouse following the injection of pTomo-sh*Nf1*;sh*p53* virus into the right sciatic nerve.

Supplemental Table 1. Current MPNST murine models

Mouse Model	Tumor phenotype	Genetic Background	Reference
Nf1$^{+/null}$; *Trp53*$^{+/null}$ cis	MPNSTs in 30% mice, 5 months	129sv x C57BL/6	[15, 16]
Nf1$^{flox/flox}$; *Ink4aArf* $^{flox/flox}$, adenoviral Cre injected into the sciatic nerves	MPNSTs in almost all mice by 4-6 months	*Nf1*$^{flox/flox}$: 129sv x C57BL/6<br>*Ink4a*$^{flox/flox}$: FVB	[19]
Nf1$^{flox/flox}$; *Dhh*-Cre; Cnp-*EGFR*	MPNSTs in 33% mice, 4-8 months	Cnp-*EGFR*: C57BL/6<br>*Nf1*$^{flox/flox}$; *Dhh*-Cre: 129sv x C57BL/6	[17]
Trp53^{R270H}; Cnp-*EGFR*	MPNSTs in 18% mice, median 284 days	Cnp-*EGFR*: FVB/N	[7]
P_0-GGFβ3 transgenic	MPNSTs 70%, 6-10 months	backcrossed to C57BL/6J x SJL/J F1	[43, 45]
P_0-GGFβ3 inbred<br>P_0-GGFβ3; *Nf1*$^{+/null}$<br>P_0-GGFβ3; *Trp53*$^{+/null}$	No tumors<br>No tumors<br>MPNSTs in 95% mice, 226 days	C57BL/6J	[44]
GFAP-Cre; *Pten*$^{flox/+}$*; LSL-Kras*$^{G12D/+}$	MPNSTs in 100% mice, 4 months	C57BL/6 x 129sv x BALB/c	[18]
Nf1$^{+/null}$; *p19Arf* $^{+/null}$	MPNSTs in 10% mice, 36 weeks	*Nf1*$^{+/null}$: 129sv<br>*p19Arf* $^{+/null}$: 129svJ6 x C57BL/6	[20]

Supplemental Table 2. Antibodies used

Antibody	Dilution	Antigen Retrieval	Secondary Antibody
S100β (ab41548; Abcam)	1:250	Citrate buffer	Rabbit
Ki67 (790-4286; Ventana)	Pre-diluted	CC1 buffer (950-124, Ventana)	Rabbit
Tryptase (760-4276; Ventana)	Pre-diluted	CC1 buffer (950-124, Ventana)	Mouse
Col4A (ab6586; Abcam)	1:1000	Citrate buffer	Rabbit
